# Associations of genetically predicted vitamin D status and deficiency with the risk of carotid artery plaque: a Mendelian randomization study

**DOI:** 10.1038/s41598-024-64731-z

**Published:** 2024-06-26

**Authors:** Devendra Meena, Marie-Joe Dib, Jingxian Huang, Alexander Smith, Jian Huang, Amrit S. Lota, Sanjay K. Prasad, Dipender Gill, Abbas Dehghan, Ioanna Tzoulaki

**Affiliations:** 1 Department of Epidemiology and Biostatistics, School of Public Health, Imperial College London, London, UK; 2 Division of Cardiovascular Medicine, Hospital of the University of Pennsylvania, Philadelphia, USA; 3 Singapore Institute for Clinical Sciences (SICS), Agency for Science, Technology and Research (A*STAR), Singapore City, Singapore; 4 Cardiovascular Magnetic Resonance Unit, Royal Brompton Hospital, Sydney St, London, SW3 6NP UK; 5 British Heart Foundation Centre of Excellence, Imperial College London, London, UK; 6 Dementia Research Centre, Imperial College London, London, UK; 7 Centre for Systems Biology, Biomedical Research Foundation, Academy of Athens, Athens, Greece

**Keywords:** Genetic association study, Cardiovascular diseases, Risk factors, Genetics research, Epidemiology

## Abstract

Low concentrations of circulating 25-hydroxy-vitamin D are observationally associated with an increased risk of subclinical atherosclerosis and cardiovascular disease. However, randomized controlled trials have not reported the beneficial effects of vitamin D supplementation on atherosclerotic cardiovascular disease (ASCVD) outcomes. Whether genetically predicted vitamin D status confers protection against the development of carotid artery plaque, a powerful predictor of subclinical atherosclerosis, remains unknown. We conducted a two-sample Mendelian randomization (MR) study to explore the association of genetically predicted vitamin D status and deficiency with the risk of developing carotid artery plaque. We leveraged three genome-wide association studies (GWAS) of vitamin D status and one GWAS of vitamin D deficiency. We used the inverse-variance weighted (IVW) approach as our main method, and MR-Egger, weighted-median, and radialMR as MR sensitivity analyses. We also conducted sensitivity analyses using biologically plausible genetic instruments located within genes encoding for vitamin D metabolism (*GC*, *CYP2R1*, *DHCR7*, *CYP24A1*). We did not find significant associations between genetically predicted vitamin D status (Odds ratio (OR) = 0.99, *P* = 0.91) and deficiency (OR = 1.00, *P* = 0.97) with the risk of carotid artery plaque. We additionally explored the potential causal effect of vitamin D status on coronary artery calcification (CAC) and carotid intima-media thickness (cIMT), two additional markers of subclinical atherosclerosis, and we did not find any significant association (β_CAC_ = − 0.14, *P* = 0.23; β_cIMT_ = 0.005, *P* = 0.19). These findings did not support the causal effects of vitamin D status and deficiency on the risk of developing subclinical atherosclerosis.

## Introduction

Circulating 25-hydroxyvitamin D, or 25(OH)D, is a reliable biomarker of vitamin D status, an essential nutrient that can be obtained through exposure to ultraviolet light, dietary intake and supplementation. Beyond its established role in bone and calcium metabolism, vitamin D has emerged as an important factor in cardiovascular health^1^. Vitamin D deficiency and insufficiency have been associated with subclinical atherosclerosis, which is in turn associated with an increased risk of cardiovascular events^2,3^. Associations between circulating 25(OH)D and carotid artery plaque, coronary artery calcification (CAC) and carotid intima-media thickness (cIMT), markers of subclinical atherosclerosis, have also been reported^4,5^. However, whether these associations are causal remains unknown. The prevalence of vitamin D deficiency (defined as serum < 20 ng/mL) worldwide is high (40% in Europe, 24% in the United States, 37% in Canada)^6^, and there is a need to better understand the clinical and subclinical consequences of vitamin D status and deficiency.

Mendelian randomization (MR) is a statistical method that employs genetic variation as an instrumental variable to assess the causal relationship between exposures and outcomes of interest. MR is analogous to a randomized controlled experiment whereby genetic variants are randomly allocated to offspring at conception. This approach mitigates the risk of confounding and reverse causality that are commonly found in traditional epidemiological studies. Previous MR studies have shown that vitamin D deficiency may play a causal role in the development of hypertension^7,8^, a known risk factor of cardiovascular disease (CVD), while others have reported limited evidence supporting a causal effect on cardiovascular traits^9,10^. However, carotid artery plaque, a measure of subclinical atherosclerosis, has not been considered as an outcome of interest, despite being a powerful predictor of future cardiovascular risk, including myocardial infarction and ischemic events^11^. Leveraging large-scale genetic data on vitamin D status and deficiency within an MR framework provides a unique opportunity to overcome some limitations of traditional epidemiological designs and RCTs and assess the effect of vitamin D on one of the most sensitive and specific predictors of ASCVD.

In this study, we harness the largest vitamin D and carotid artery plaque GWAS to date to assess the potential causal effect of vitamin D status and deficiency on the risk of carotid artery plaque using a two-sample MR study design. As secondary analyses, we further explore the potential causal effect of vitamin D status on CAC and cIMT, as they had also been considered important markers for the development of atherosclerosis^12,13^.

## Methods

We used a two-sample MR design to assess the associations between genetically predicted vitamin D status and deficiency and risk of carotid artery plaque. For MR causal estimates to be valid, the following assumptions must be met: the genetic instruments (1) are strongly associated with the exposure, (2) are not associated with any potential confounder of the exposure–outcome association, and (3) do not affect outcome independently of the exposure^9^. Summary-level genetic association data were obtained from publicly available GWAS and are outlined in Table 1. Each study obtained ethical approval and participant consent.

**Table 1:** Data sources used for two-sample Mendelian randomization (MR) analyses.

Trait	IEU GWAS database ID (IGD)	Sample size	Year	Author	PMID
Vitamin D levels	ebi-a-GCST005367	79,366	2018	Jiang et al.	29343764
Serum 25-hydroxyvitamin D levels	ebi-a-GCST90000618	417,580	2020	Revez et al.	32242144
25 Hydroxyvitamin D level	ieu-b-4812	441,291	2020	Manousaki et al.	32059762
Vitamin D deficiency	finn-b-E4_VIT_D_DEF	209,789	2021	FinnGen consortium	NA
Carotid artery plaque	NA	48,434	2018	Franceschini et al.	30510157
Coronary artery calcification	ebi-a-GCST90278456	26,909	2023	Kavousi M. et al.	37770635
Carotid intima-media thickness	NA	48,434	2018	Franceschini et al.	30510157

*IEU* Integrative Epidemiology Unit.

### Genetic predictors of vitamin D status

We obtained genetic estimates for circulating 25-hydroxyvitamin D concentrations from three different sources (Table 1). The first is a GWAS of 417,580 individuals of European ancestry in the UK Biobank^14^ (ID: ebi-a-GCST90000618; https://gwas.mrcieu.ac.uk/datasets/ebi-a-GCST90000618/). Circulatory levels of 25(OH)D were measured in blood samples using a chemiluminescent immunoassay that measures total concentrations of 25(OH)D (25(OH)D_3_ and 25(OH)D_2_). The second GWAS was performed in the UK Biobank population^15^ (N = 441,291; ID: ieu-b-4812; https://gwas.mrcieu.ac.uk/datasets/ieu-b-4812/). Circulatory 25(OH)D levels (nmol/L) were measured using the Diasorin assay. For 6% of the study cohort, adjustments were made by subtracting 21.2 nmol/L from the measurements in the vitamin D supplement users. Further adjustments are described fully in the original study^16^. The third GWAS was a metaGWAS study of 31 studies with a total of 79,366 individuals of European descent^17^ (ID: ebi-a-GCST005367; https://www.ebi.ac.uk/gwas/studies/GCST005367).

### Genetic predictors of vitamin D deficiency

We obtained summary statistics from the FinnGen biobank (ID: finn-b-E4_VIT_D_DEF; https://gwas.mrcieu.ac.uk/datasets/finn-b-E4_VIT_D_DEF/) on vitamin D deficiency from N_cases_ = 182 and N_controls_ 209,607 (Table 1). Further details on the endpoint definition can be found at https://risteys.finregistry.fi/endpoints/E4_VIT_D_DEF (ICD-10 E50-E64)^18^.

### GWAS summary statistics of carotid artery plaque cIMT, and CAC

Genetic associations of carotid artery plaque and cIMT were obtained from a meta-analysis of genome-wide association study (GWAS) from 17 studies from the Cohorts for Heart and Aging Research in Genomic Epidemiology (CHARGE) consortium and the University College London-Edinburgh-Bristol (UCLEB) consortium^19^ (Table 1, available at https://www.ncbi.nlm.nih.gov/projects/gap/cgi-bin/study.cgi?study_id=phs000930.v6.p1). A total of 48,434 individuals of European ancestry were included in the analysis, 21,540 of which had carotid artery plaque defined by atherosclerotic thickening of the common carotid artery wall or the proxy measure of luminal stenosis greater than 25%^19^. Carotid artery plaque was considered a dichotomous trait while cIMT was considered a continuous trait. Summary statistics of CAC were obtained from a GWAS included 26,909 individuals of European ancestry from16 cohorts^20^. CAC was measured employing computed tomography and was considered a continuous trait.

### Selection of genetic instruments

We selected SNPs that were associated with serum vitamin D levels at p < 5 × 10^−8^ and r^2^ = 0.001. A less stringent p-value threshold (p < 5 × 10^−6^) was used for the vitamin D deficiency GWAS from FinnGen since no SNP reached genome-wide significance. To minimize weak instrument bias, we only selected SNPs that had F-statistics > 10. Additionally, we retrieved SNPs located within four key genes involved in the vitamin D synthesis pathway, namely the vitamin D binding protein (*GC*), 25-OH hydroxylase (*CYP2R1*), 7-dehydrocholesterol reductase (*DHCR7*), and 24-hydroxylase (*CYP24A1*) using the UCSC MySQL server. The summary level data for these SNPs were subsequently extracted from the largest GWAS of vitamin D status to calculate MR estimates (P < 5 × 10^−8^ and r^2^ < 0.001)^14^.

### Two-sample MR analyses

SNP-specific causal effects for the selected genetic instruments were estimated using Wald ratio, i.e., SNP-outcome association divided by SNP-exposure association^21^. To obtain the MR effect estimates, we pooled the SNP-specific estimates using inverse-variance weighted (IVW) random effects model as the main method^22^. We used weighted median and MR-Egger regression as sensitivity methods to assess the robustness of IVW estimates and horizontal pleiotropic effects^23,24^. Potential outlier SNPs identified using MR-PRESSO and radial regression were excluded from the analysis^25,26^. To orient the direction of causality, we applied Steiger filtering, which excludes the SNPs with a larger variance explained in the outcome than in the exposure^27^. We also applied Radial MR to detect potential outliers within MR analysis^26^.

## Results

**Figure 1:**
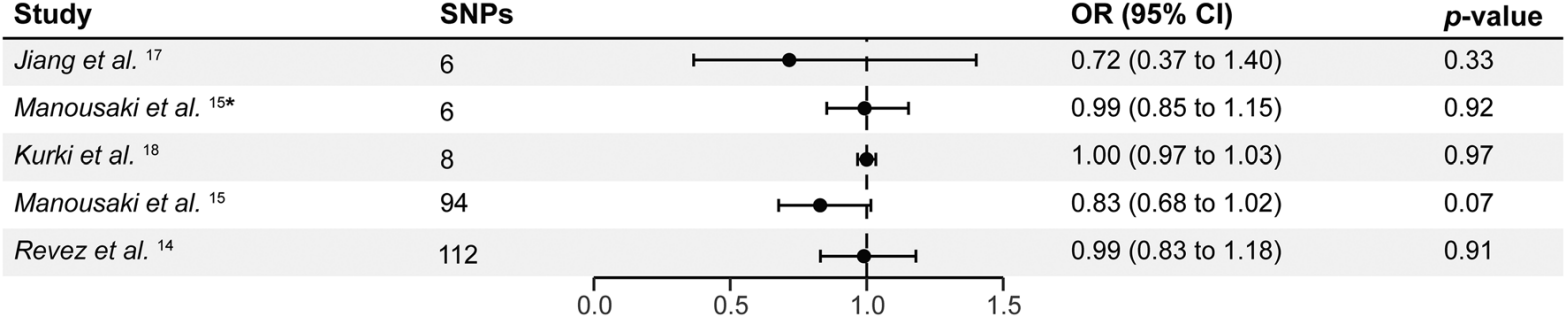
The effects of genetically predicted vitamin D status and deficiency on the risk of carotid artery plaque in different studies. Estimates illustrated in this plot represent IVW estimates. Estimates are shown as odds ratios. Horizontal lines represent the 95% CIs. *SNPs* single nucleotide polymorphism, *CI* confidence intervals, *OR* odds ratio. * represents SNPs that have a known biological role in vitamin D synthesis and metabolism.

**Figure 2:**
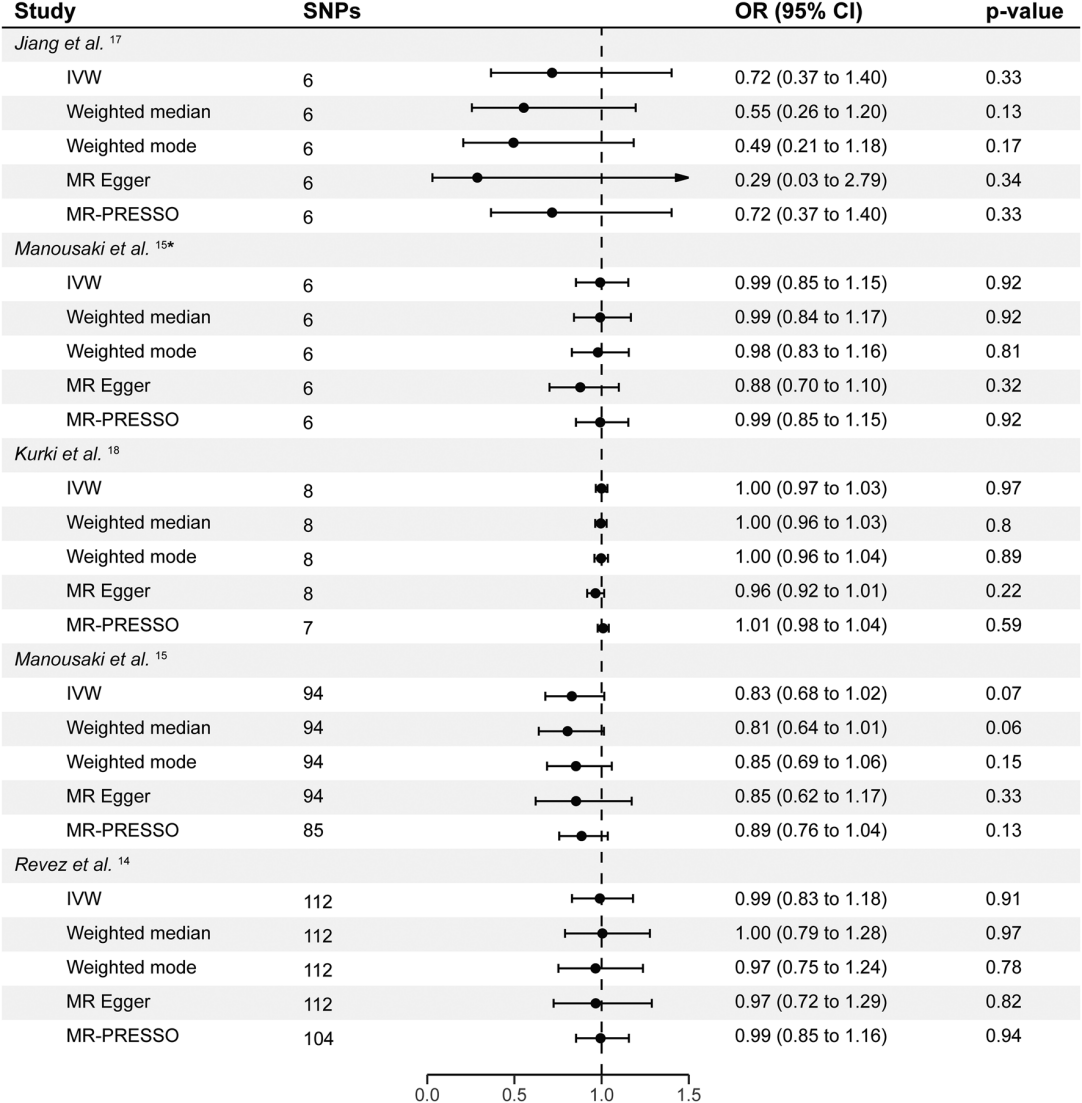
MR analyses of the effect of genetically predicted vitamin D status and deficiency on the risk of carotid artery plaque. Estimates are shown as odds ratios. Horizontal lines represent the 95% CIs. SNPs: number of SNPs used for the estimation of the causal effects in each model. *SNPs* single nucleotide polymorphism, *CI* confidence intervals, *OR* odds ratio. * represents SNPs that have a known biological role in vitamin D synthesis and metabolism.

**Figure 3:**
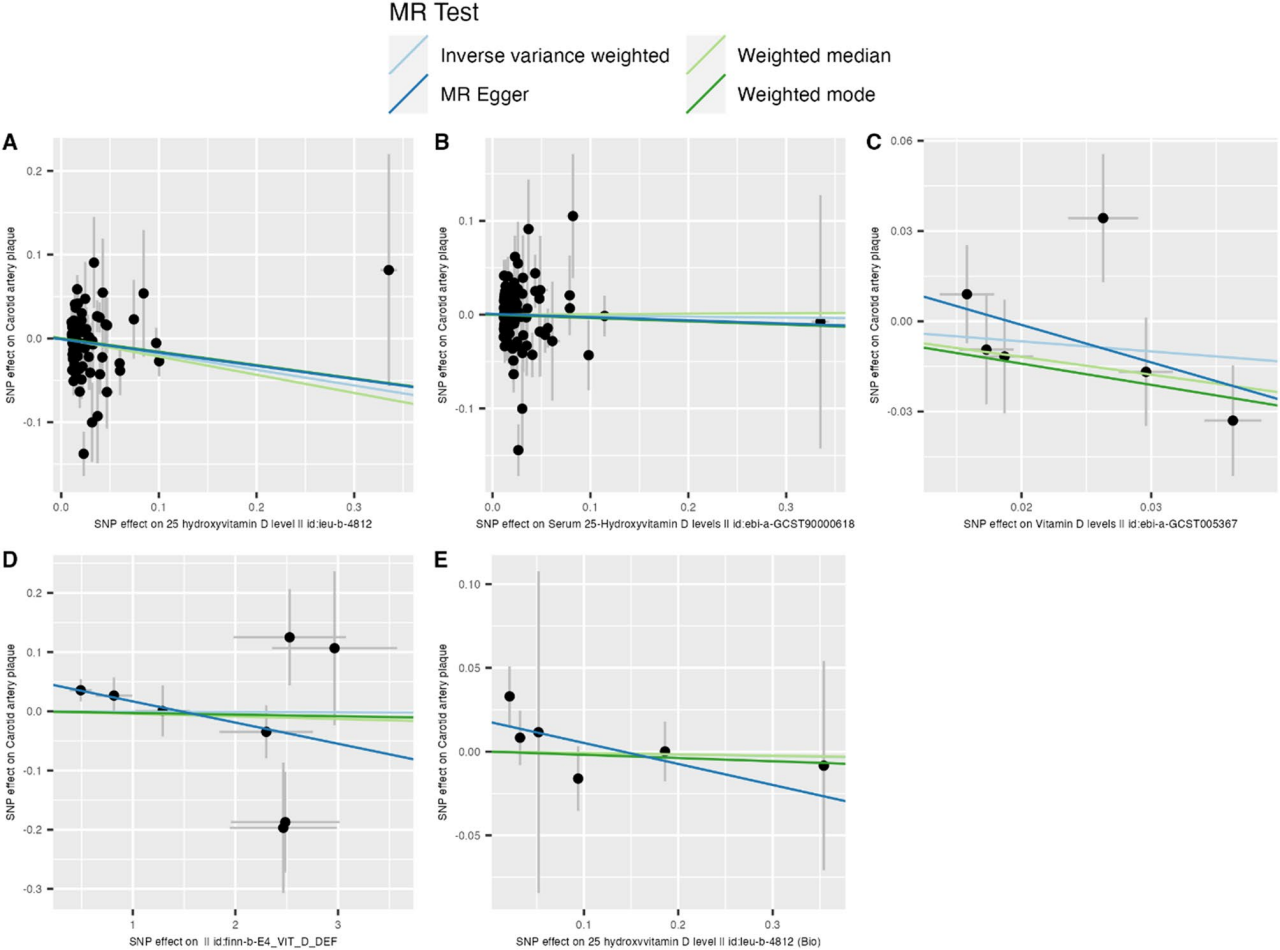
Scatter plots for MR analyses of the causal effect of genetically predicted vitamin D status and deficiency on the risk of carotid artery plaque. The slope of each line corresponds to the estimated MR effect per method. (**A**) ieu_b_4812; (**B**) ebi_a_GCST90000618; (**C**) ebi_a_GCST005367; (**D**) finn-b-E4_VIT_D_DEF; (**E**) ieu_b_4812_bio.

The number of uncorrelated SNPs associated with vitamin D status and deficiency ranged from 6 to 112 (Figs. 1, 2 and Supplementary Table 1). The mean F statistics ranged from 22.52 to 2179 indicating no weak instrument bias. Our analysis did not reveal any substantial evidence supporting an effect of vitamin D on carotid artery plaque using 94 vitamin D SNPs from the largest GWAS (OR_IVW_ = 0.83, P_IVW_ = 0.07, 95% CI = 0.68 to 1.02; Figs. 1, 2, 3, Supplementary Table 1). Sensitivity methods showed a consistent direction of effect and MR-Egger intercept did not suggest any evidence of horizontal pleiotropy (P_Egger_intercept_ = 0.81). There was evidence of substantial heterogeneity among the individual SNP effect estimates as indicated by the Cochran *Q* test (Q_pval < 0.001; Supplementary Table 1). MR-PRESSO and IVW radial regression identified 9 SNPs as potential outliers (Supplementary Fig. 4, Supplementary Table 2). However, the IVW estimates after removing outliers showed no evidence of an effect of vitamin D on carotid artery plaque (OR_IVW_ = 0.89, P_IVW_ = 0.13, 95% CI = 0.76 to 1.04; Supplementary Table 2). Steiger filtering did not identify SNPs that showed a stronger association with carotid artery plaque than with vitamin D (OR_IVW_ = 0.89, P_IVW_ = 0.13, 95% CI = 0.76 to 1.04; Supplementary Table 2). IVW estimates calculated for vitamin D status and deficiency using the three other GWAS datasets were non-significant (OR_IVW_ range = 0.72 to 1.00, P_IVW_ > 0.05). Sensitivity methods showed a consistent direction of effect for each GWAS used (Fig. 2, Supplementary Table 1). While no evidence of horizontal pleiotropy was found, we did observe significant heterogeneity when utilizing SNP-specific estimates from Revez et al.^14^ (Supplementary Table 1). The IVW estimates remained non-significant after excluding outliers from Revez et al.^14^ and FinnGen GWAS^18^ (Supplementary Table 2).

The IVW estimates, derived from 6 SNPs chosen due to their involvement in vitamin D synthesis and metabolism, did not reveal a significant association between vitamin D and carotid artery plaque (OR_IVW_ = 0.99, P_IVW_ = 0.92, 95% CI = 0.85 to 1.15; Figs. 1, 2, Supplementary Table 1). There was no evidence for substantial heterogeneity among the individual SNP effect estimates calculated using the Cochran *Q* test (Q_pval > 0.05; Supplementary Table 1). The harmonized dataset used to estimate the MR estimate from each exposure is available in Supplementary Tables 3–6. Single SNP effect estimates, Leave-one-out effect estimates, and their respective forest plots can be found in the Supplementary Tables 8–12 and Supplementary Figs. 1–3.

**Figure 4:**
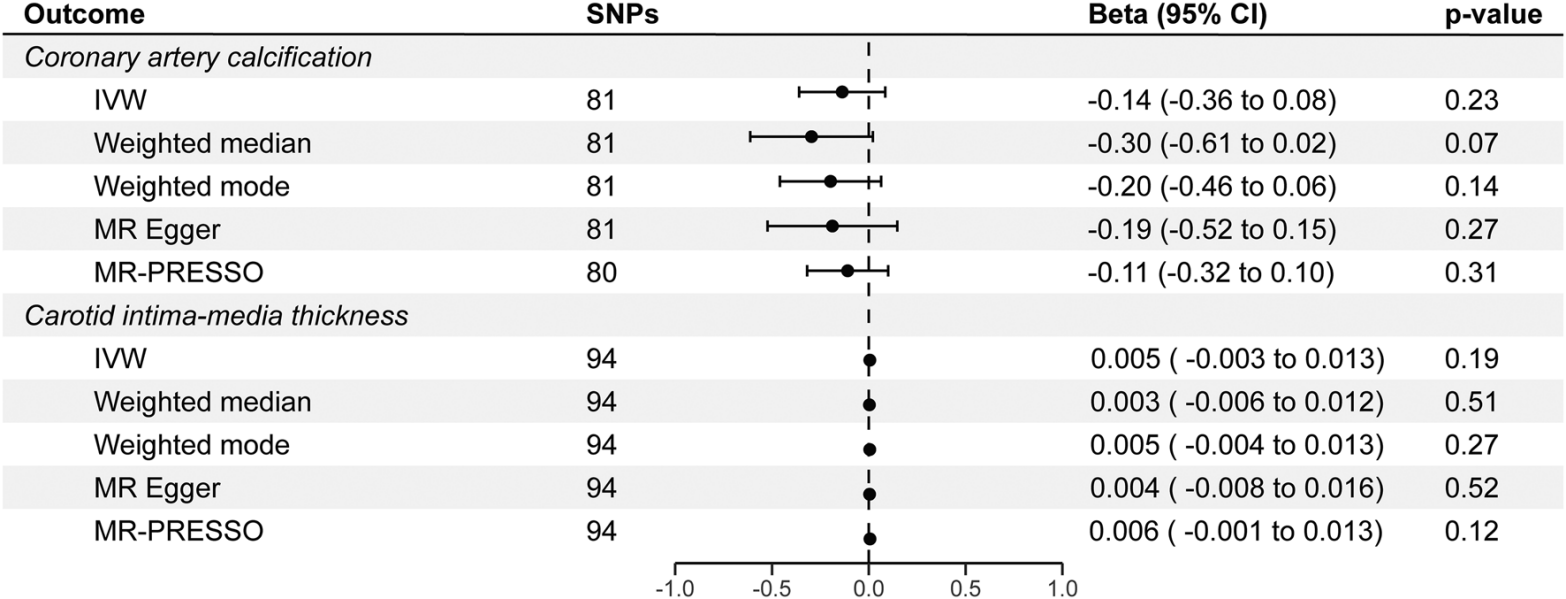
MR analyses of the effect of genetically predicted vitamin D status on coronary artery calcification and carotid intima-media thickness. Estimates are shown as beta. Horizontal lines represent the 95% CIs. SNPs: number of SNPs used for the estimation of the causal effects in each model. *SNPs* single nucleotide polymorphism, *CI* confidence intervals.

By leveraging the GWAS of vitamin D status with the largest sample size in our primary analyses, we did not find a significant association between vitamin D and cIMT (β_IVW_ = 0.005, P_IVW_ = 0.19, 95% CI = − 0.003 to 0.013), and CAC (β_IVW_ = − 0.14, P_IVW_ = 0.23, 95% CI = − 0.36 to 0.08; Fig. 4, Supplementary Table 13). The estimates were consistent across sensitivity methods and there was no indication of horizontal pleiotropy (P_Egger_intercept cIMT_ = 0.79, P_Egger_intercept CAC_ = 0.69). Potential heterogenous SNPs were identified by MR-PRESSO and IVW radial regression, however, no significant association was revealed after the removal of these outliers (Supplementary Fig. 5, Supplementary Table 14). Likewise, the results remain unchanged after the removal of variants that demonstrated potential reverse causality in Steiger filtering (Supplementary Table 14). Related MR scatter plots and funnel plots were shown in Supplementary Figs. 6, 7. The harmonized dataset used in MR analyses for CAC and cIMT is available in Supplementary Tables 15, 16. Single SNP effect estimates, Leave-one-out effect estimates, and their respective forest plots can be found in the Supplementary Tables 17, 18 and Supplementary Figs. 8, 9.

## Discussion

To our knowledge, this is the first study to investigate the associations between genetically predicted vitamin D status and deficiency and carotid artery plaque risk using a two-sample MR approach. Our analyses indicated that lower genetically predicted vitamin D status and deficiency were not associated with a greater risk of carotid artery plaque. Furthermore, we did not find a significant association between genetically predicted vitamin D status and cIMT, and CAC. These findings suggest that vitamin D supplementation is not likely to be beneficial in remedying carotid artery plaque in populations that are vitamin D deficient nor those who may meet the requirement for optimal vitamin D status. Our findings suggest that vitamin D supplementation may not be conclusively beneficial in reducing carotid artery plaque, both in populations with vitamin D deficiency and those meeting optimal vitamin D status, prompting the need for further investigation in well-designed RCTs taking carotid artery plaque as the primary endpoint in future studies.

The development of atherosclerotic plaque is influenced by multiple biological processes which may be a potential explanation for this finding. The role of vitamin D in modulating immune responses, inflammation and vascular health is well-established^28,29^. Therefore, vitamin D may affect carotid artery plaque formation through any of these mechanisms rather than having a direct effect. This is confirmed through our MR findings, whereby the removal of heterogeneous SNPs led to the attenuation of our findings. Another explanation may be that current GWAS on vitamin D status did not distinguish between the circulating form of vitamin D (25(OH)D) and its active form, 1,25-dihydroxycholecalciferol, which is converted by the enzyme 25-hydroxyvitamin D3 1-alpha-hydroxylase (CYP27B1) in kidney^30^. Moreover, the time frame and dosage of vitamin D exposure may be critical factors influencing carotid artery plaque formation. Our study primarily focused on genetically predicted vitamin D status, overlooking the potential variations in the duration and intensity of vitamin D exposure over a lifetime. Additionally, evidence suggests that there may be a non-linear association between circulating vitamin D levels and the risk of CVD^31,32^. This may also be the case for the association between circulating vitamin D and the risk of carotid artery plaque that we could not test in the current study due to methodological challenges^33,34^. Subsequent investigations may employ a non-linear MR framework to scrutinize this association more comprehensively.

Our findings add to the existing empirical evidence of the effects of vitamin D supplementation on cardiovascular health and disease. Previous RCTs have shown that vitamin D supplementation did not influence blood pressure and cardiovascular risk. However, it was associated with decreased ApoB concentration and pulse wave velocity, a marker of arterial stiffness^35^. Results from previous MR studies are conflicting, potentially due to the heterogeneity in characteristics of the populations under study. Huang et al. did not identify significant associations between genetically predicted vitamin D and the risk of ischaemic cardiovascular events in Europeans and Chinese adults^36^. Similarly, other MR studies have reported null findings for associations of genetically predicted vitamin D status on the risk of ischemic heart disease and cardiovascular mortality^37–39^.

Previous RCTs assessing the role of vitamin D in cardiovascular health did not include individuals with vitamin D deficiency or subgroups in their study designs. Therefore, there is not enough evidence to determine whether vitamin D deficiency plays a role in cardiovascular disease, or if it should be targeted for cardiovascular disease prevention. There is a need to evaluate the role of vitamin D supplementation on cardiovascular risk factors and outcomes in vitamin D deficiency populations by collecting more clinical and omics data on vitamin D deficient individuals in biobanks, and by carefully designing RCTs specific to this population subgroup.

### Strengths and limitations

Our study has several strengths. The utilization of genetic variants strongly associated with vitamin D status, deficiency and metabolism as instrumental variables enabled us to mitigate biases inherent to traditional epidemiological studies, including confounding and reverse causation. We also employed various sensitivity methods, including the identification of outliers, to ensure the robustness of our assumptions in the MR model. Findings were consistent across main and sensitivity analyses, thereby substantiating the validity of our findings. Our work also has several limitations. MR analyses rely on assumptions. While we rigorously assessed MR assumptions to ensure the robustness of our findings, unaccounted pleiotropy may still have potentially biased our results. Additionally, all GWAS resources used in these analyses pertain to populations of European ancestry, therefore our findings may not be generalizable to other populations and ancestries as allelic differences between ancestries may produce different effect estimates. There is a need to assess the population-specific effects of vitamin D status and deficiency on the risk of ASCVD. Additionally, causal inferences made through our MR analyses should be interpreted cautiously, as the MR paradigm measures the lifelong effect of genetic variants, and its estimates should not be directly translated to assume the effect of short-term clinical interventions on vitamin D status and deficiency. Lastly, through this MR design, we are unable to explore potential interactions between vitamin D status and deficiency-associated genetic variants and environmental factors, which may contribute to the observed effect or modify it.

## Conclusions

This is the first study to assess the causal effect of vitamin D status and deficiency on the risk of carotid artery plaque using a powerful approach that leverages different types of methodologies to ensure the most robust findings. Our findings did not provide evidence for a potentially causal relationship between genetically predicted vitamin D status and carotid artery plaque development, CAC and cIMT. Taking these findings into account with the totality of the evidence in the literature, this highlights the complexity of atherosclerosis development and underscores the need to understand the impact of vitamin D interplay with other risk factors on the risk of ASCVD. Future studies incorporating larger sample sizes of vitamin D deficiency may provide further insights into understanding the role of vitamin D deficiency in ASCVD.

### Supplementary Information

Supplementary Figures.

Supplementary Tables.

## Data Availability

All GWAS summary statistics used in this study are publicly available for download and a link was provided for each dataset in the Method section. The carotid artery plaque GWAS summary statistics data that support the findings of this study was obtained from database of Genotypes and Phenotypes (dbGaP) under the CHARGE acquisition number (https://www.ncbi.nlm.nih.gov/projects/gap/cgi-bin/study.cgi?study_id=phs000930.v6.p1; accession phs000930.v6.p1).

## Code availability

The following software packages were used for data analysis: R (https://www.r-project.org) R version 4.3.1 (2023-06-16), ‘TwoSampleMR’ version 0.5.7, ‘RadialMR’ version 1.1^26^, ‘MRPRESSO’ version 1.0^25^. The code used to run the current analysis will be made available on GitHub (https://github.com/Jingxian-Huang/vit_d_carotid_plaque_mr).
