# Supplementary material for: Associations of genetically predicted vitamin D status and deficiency with the risk of carotid artery plaque: a Mendelian randomization study
Source: Sci Rep. 2024 Jun 26;14:14743. doi: 10.1038/s41598-024-64731-z (PMC11208549; doi:10.1038/s41598-024-64731-z)
Supplement: Supplementary file 1 — Supplementary Figures. [file 41598_2024_64731_MOESM1_ESM.docx]

**Supplementary Figures**

**Associations of Genetically Predicted Vitamin D Status and Deficiency with the Risk of Carotid Artery Plaque: A Mendelian Randomization Study**

**Devendra Meena, DPhil^1†^; Marie-Joe Dib, PhD^2†^; Jingxian Huang, MSc^1†^; Alexander G. Smith, PhD^1^; Jian Huang, PhD^1,3^; Amrit S. Lota, PhD^4^; Sanjay K. Prasad, MD^4^; Dipender Gill, PhD^1^; Abbas Dehghan, MD, PhD^1,6^; Ioanna Tzoulaki, PhD^1,5,6*^**

^1^Department of Epidemiology and Biostatistics, School of Public Health, Imperial College London, London, UK

^2^Division of Cardiovascular Medicine, Hospital of the University of Pennsylvania, Philadelphia, USA

^3^Singapore Institute for Clinical Sciences (SICS), Agency for Science, Technology and Research (A*STAR), Singapore

^4^Cardiovascular Magnetic Resonance Unit, Royal Brompton Hospital, Sydney St, London, UK SW3 6NP

^5^British Heart Foundation Centre of Excellence, Imperial College London, London, UK

^6^Dementia Research Centre, Imperial College London, London, UK

***Corresponding author:** Ioanna Tzoulaki, PhD (i.tzoulaki@imperial.ac.uk)

**†Equal contribution**

**
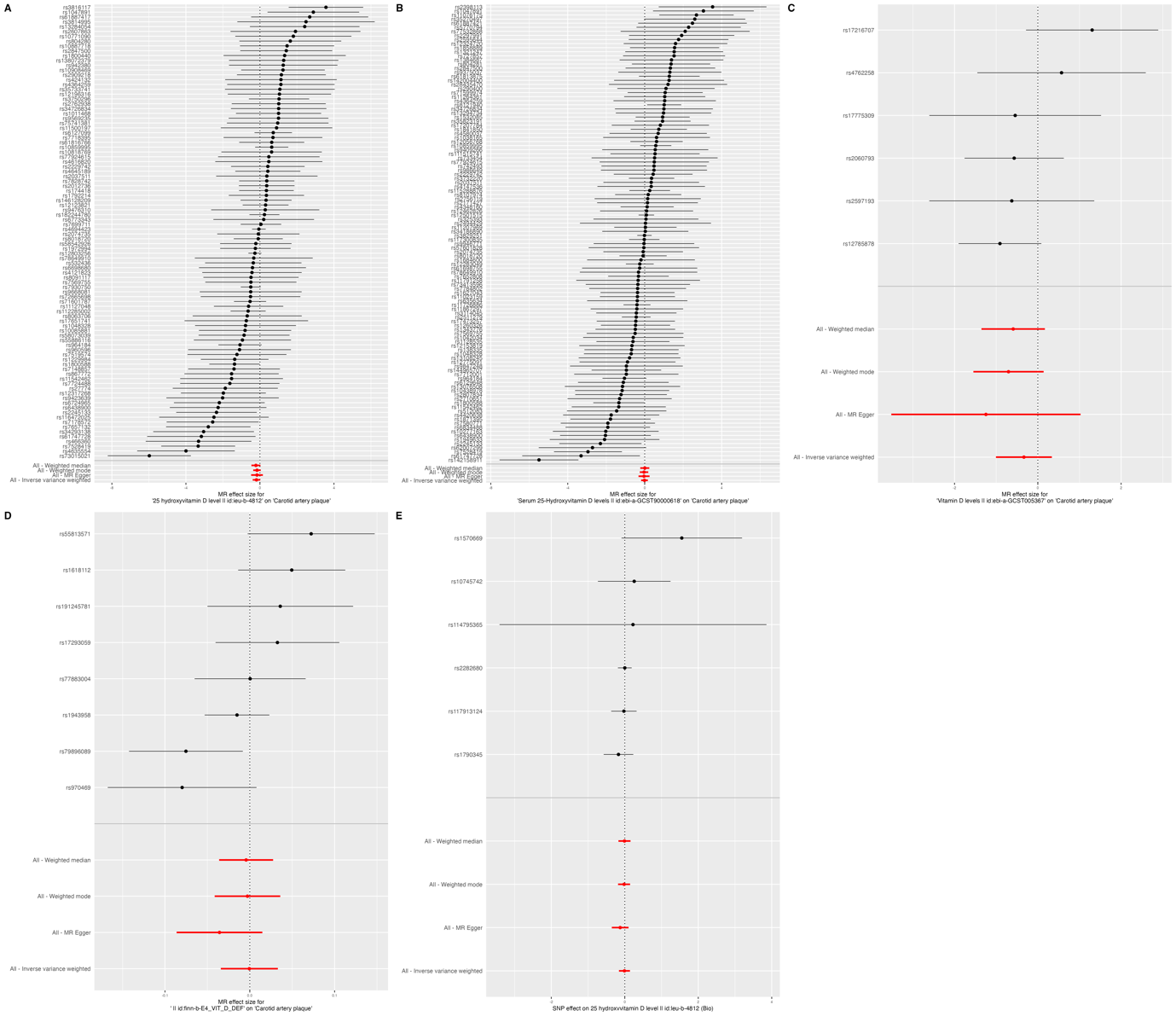
**

**Supplementary Figure 1**. Forest plots of the single and multi-SNP analyses for the SNP effect of vitamin D levels and carotid artery plaque. The x-axis shows the MR effect size for the vitamin D levels or deficiency on carotid artery plaque. The y-axis shows single-SNP (black lines) as well as multi-SNP ratio estimates (red line). Weighted median, weighted mode, and MR-Egger show sensitivity meta-analyses. Error bars indicate 95% confidence intervals. **A)** ieu_b_4812; **B)** ebi_a_GCST90000618; **C)** ebi_a_GCST005367; **D)** finn-b-E4_VIT_D_DEF; **E)** ieu_b_4812_bio.

**
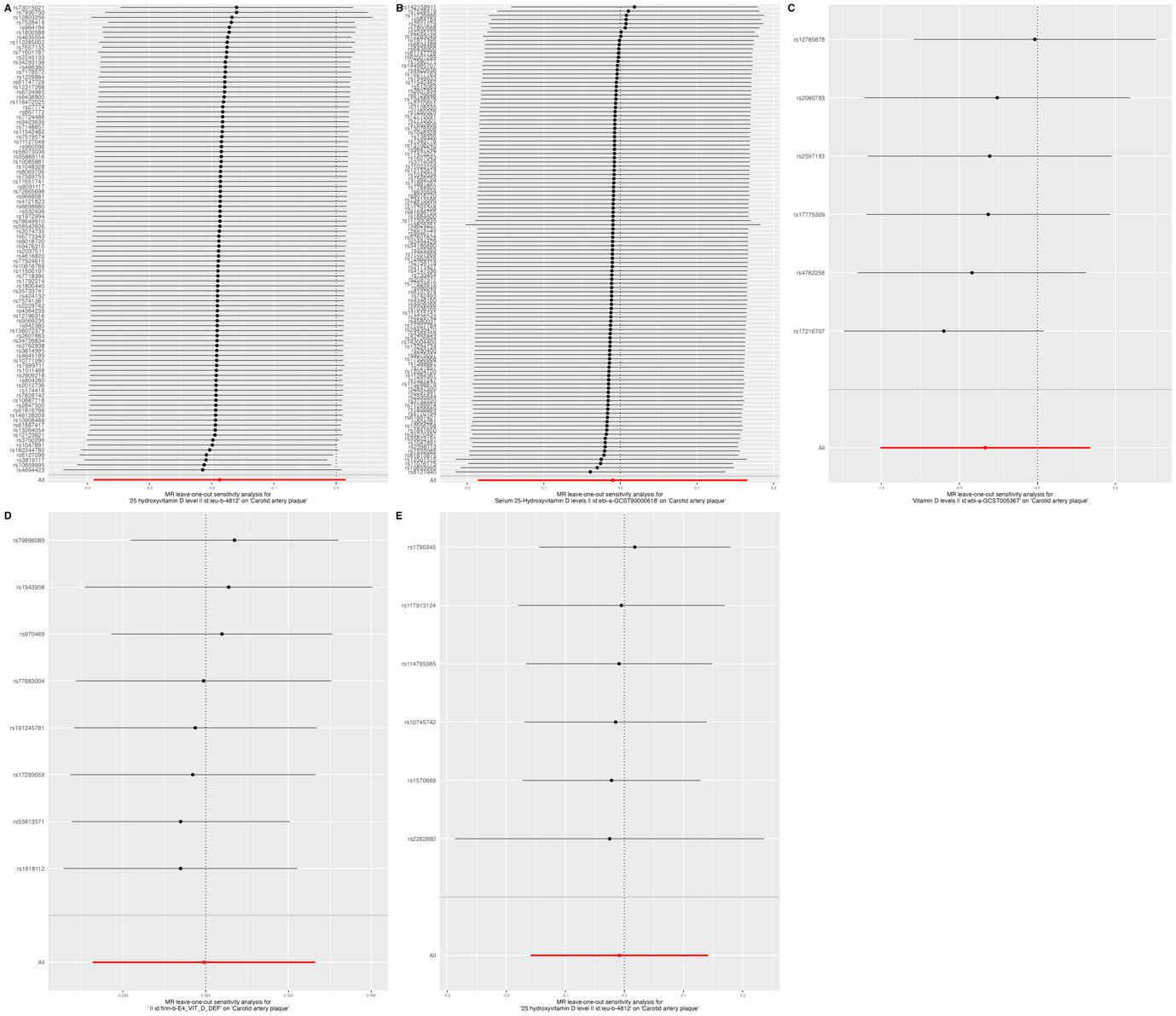
**

**Supplementary Figure 2**. Forest plot of "leave-one-out" sensitivity analysis method to show the influence of single-SNPs on the MR estimates. The black and red lines indicate single-SNP and multi-SNP analysis, respectively. Error bars indicate 95% confidence intervals. **A)** ieu_b_4812; **B)** ebi_a_GCST90000618; **C)** ebi_a_GCST005367; **D)** finn-b-E4_VIT_D_DEF; **E)** ieu_b_4812_bio

**
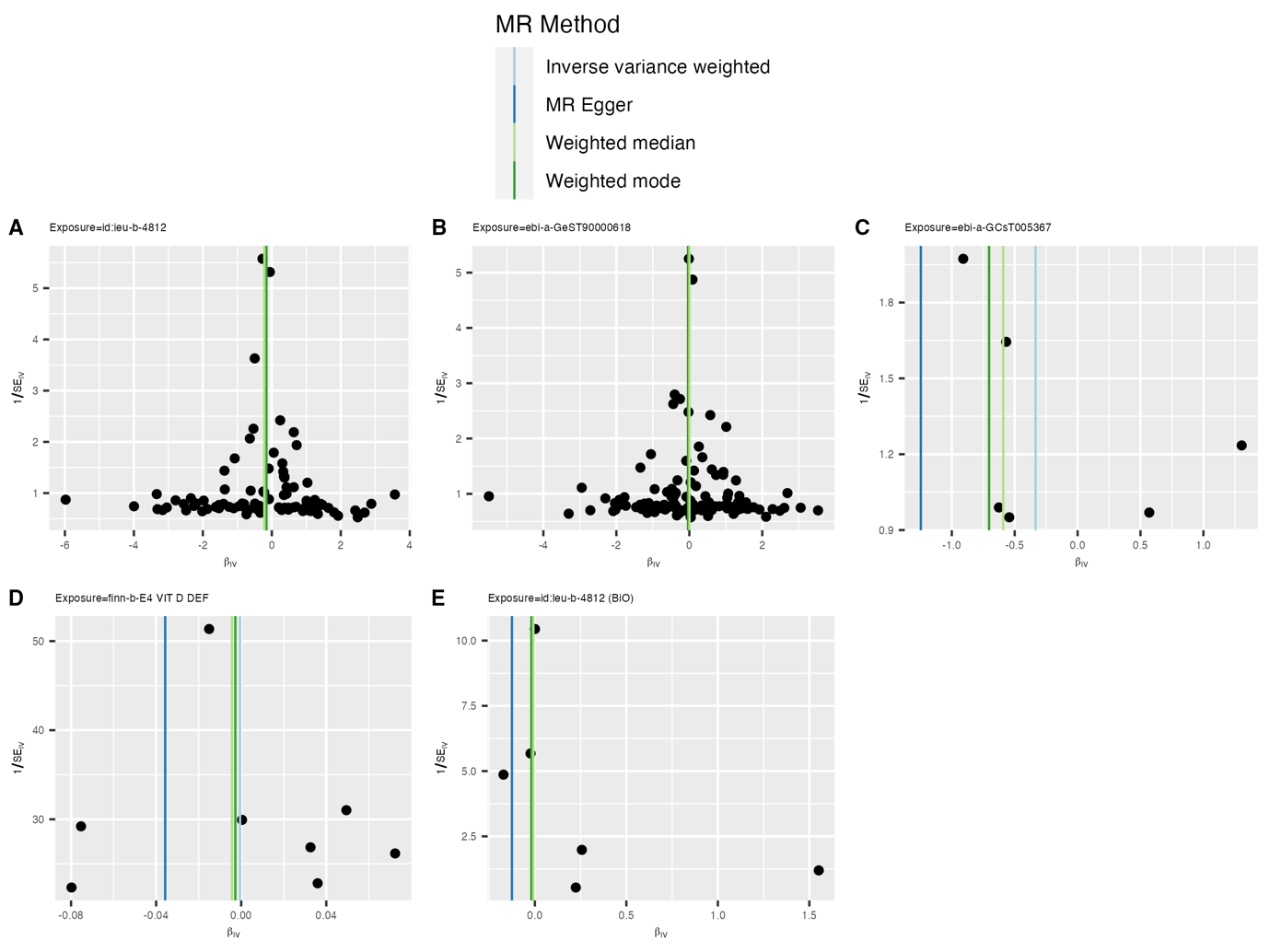
**

**Supplementary Figure 3**. Funnel plot of single-SNP effect estimates and corresponding inverse standard errors. **A)** ieu_b_4812; **B)** ebi_a_GCST90000618; **C)** ebi_a_GCST005367; **D)** finn-b-E4_VIT_D_DEF; **E)** ieu_b_4812_bio

**
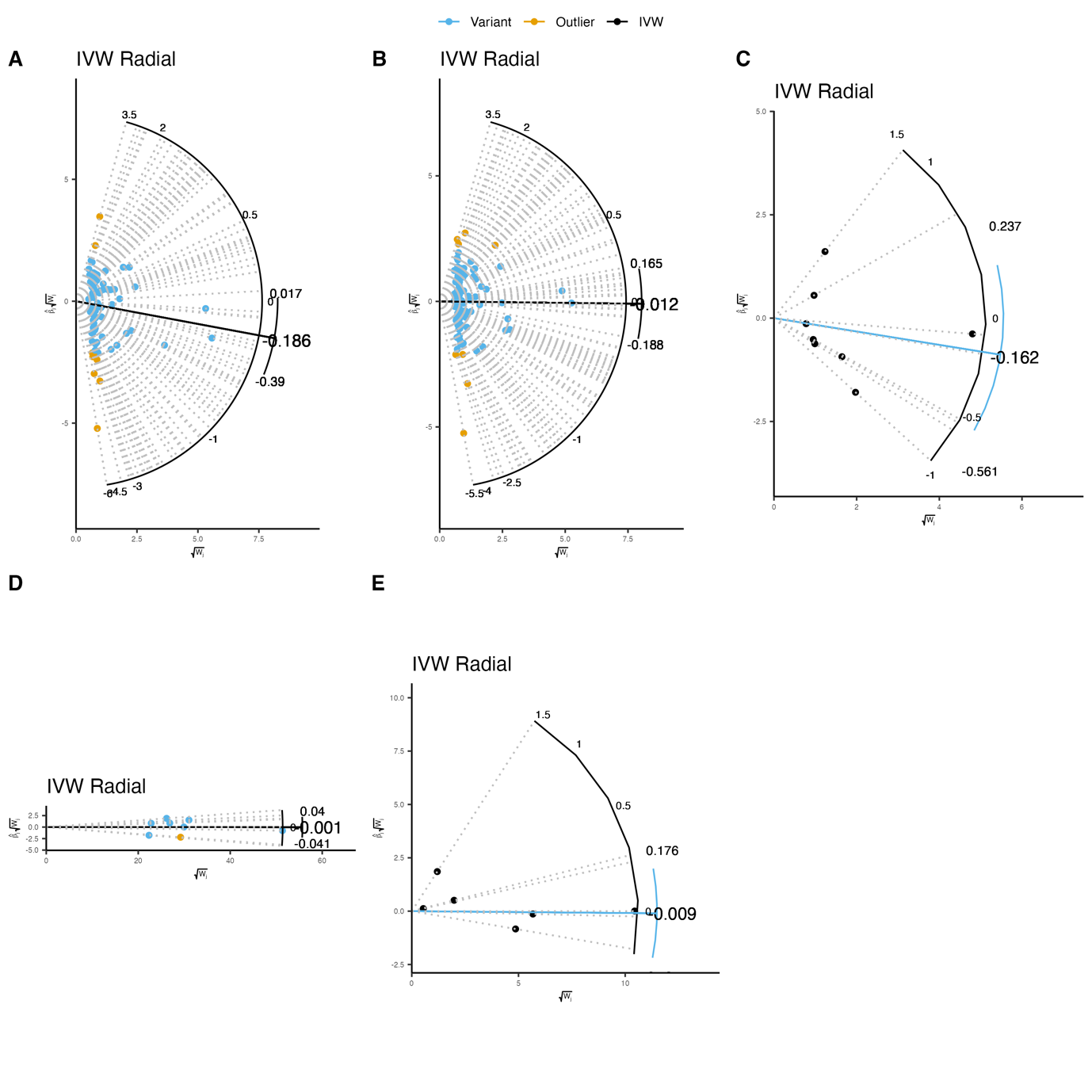
**

**Supplementary Figure 4**. Radial plots to identify potential outliers for the effect of vitamin D levels/deficiency on carotid plaque. The blue line represents the overall inverse-variance weighted estimates. Black and yellow dots show valid and outlier SNPs, respectively. **A)** ieu_b_4812; **B)** ebi_a_GCST90000618; **C)** ebi_a_GCST005367; **D)** finn-b-E4_VIT_D_DEF; **E)** ieu_b_4812_bio


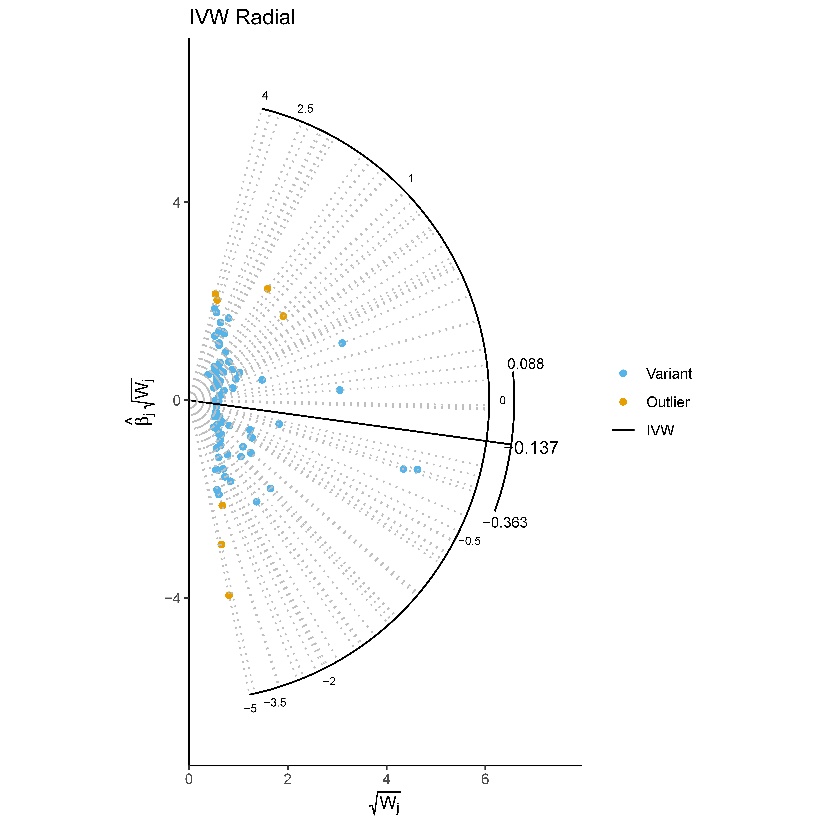
**
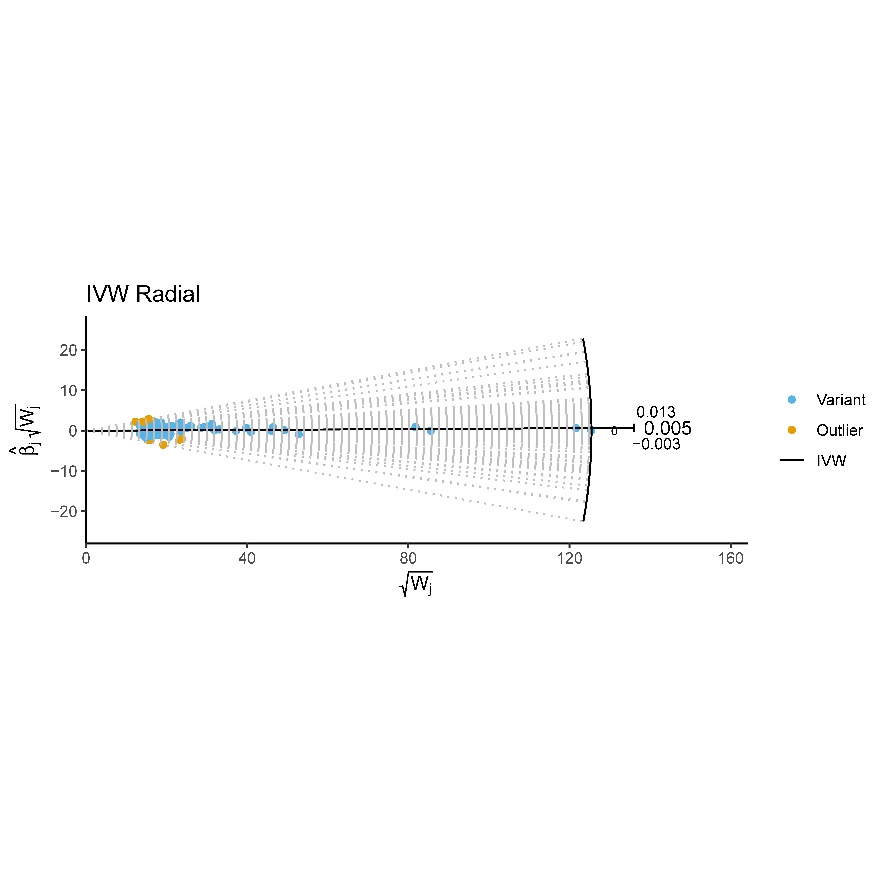
**

**B**

**A**

**Supplementary Figure 5**. Radial plots to identify potential outliers for the effect of vitamin D levels on **A)** coronary artery calcification and **B)** carotid intima-media thickness. The blue line represents the overall inverse-variance weighted estimates. Black and yellow dots show valid and outlier SNPs, respectively.


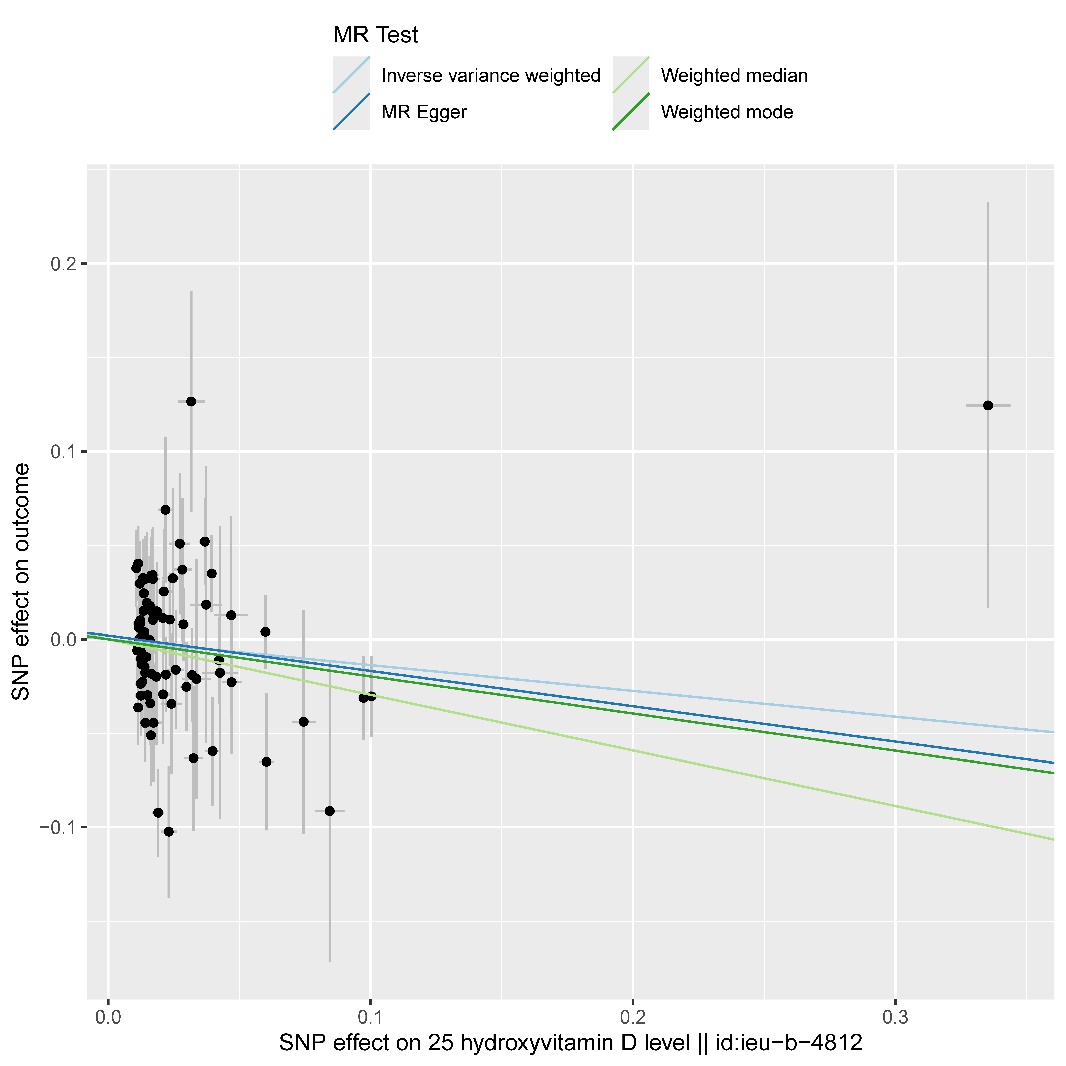


**B**

**A**


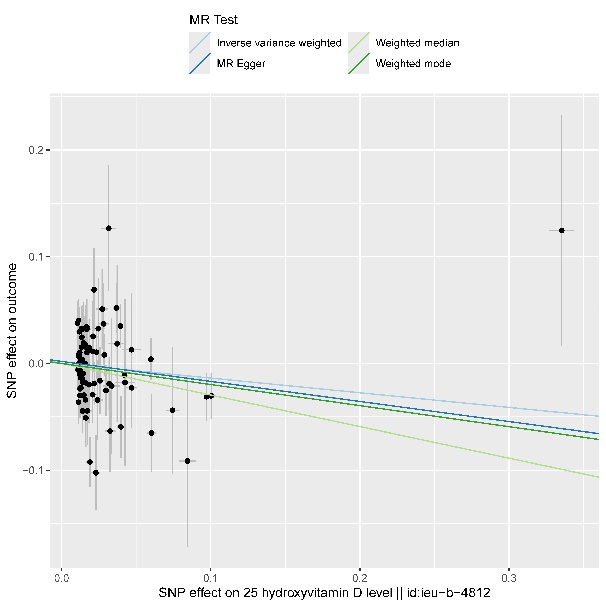

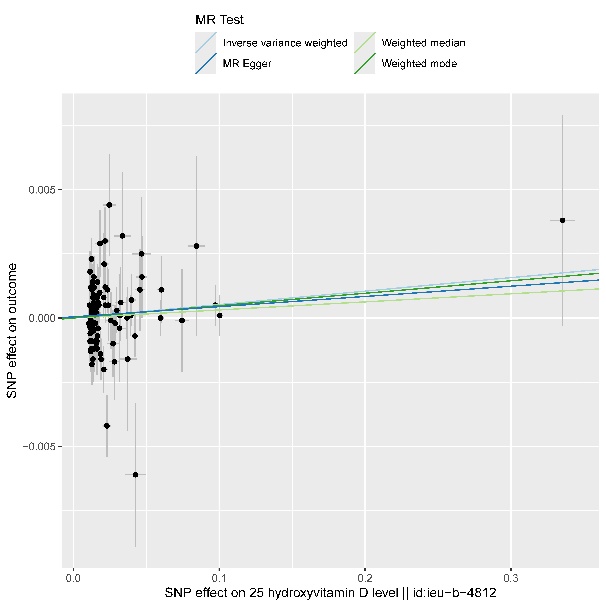


**Supplementary Figure 6**. Scatter plots for MR analyses of the causal effect of genetically predicted vitamin D status on **A)** coronary artery calcification and **B)** carotid intima-media thickness. The slope of each line corresponds to the estimated MR effect per method.

**B**


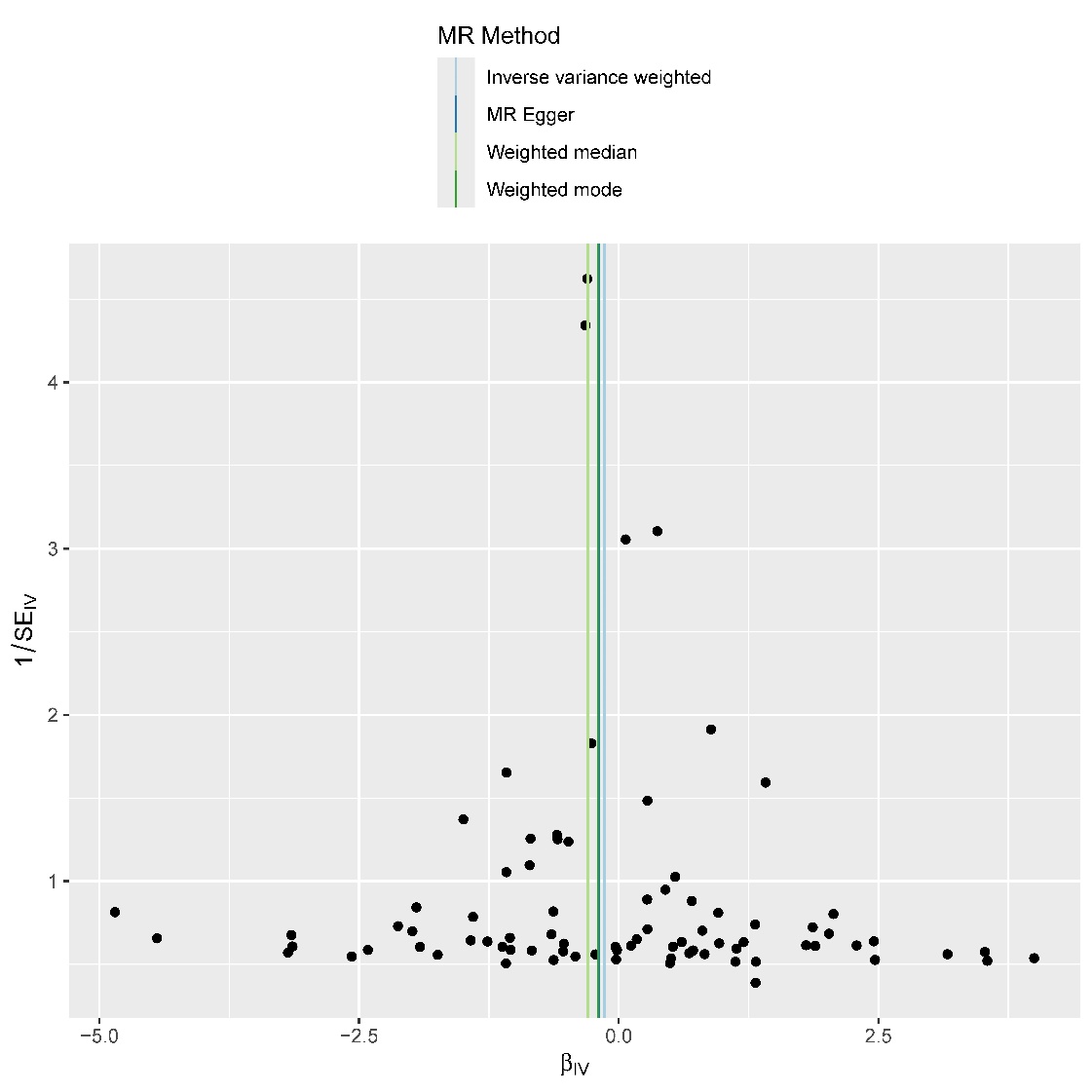


**B**

**A**


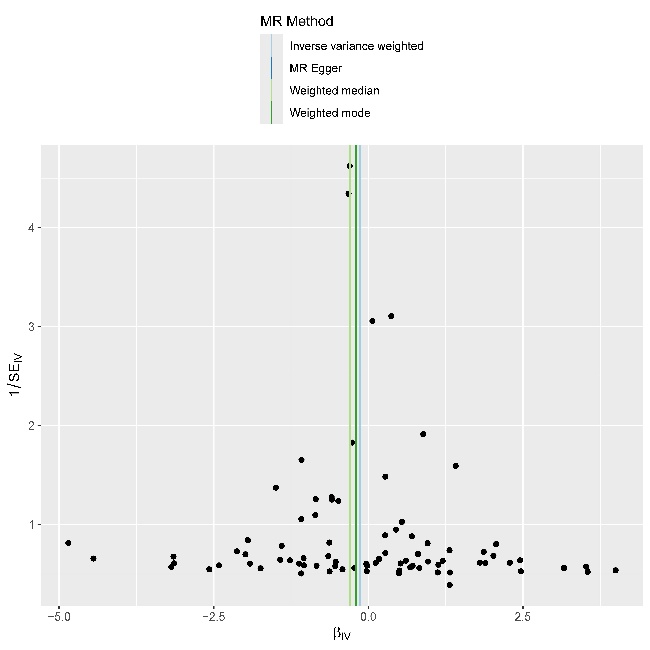

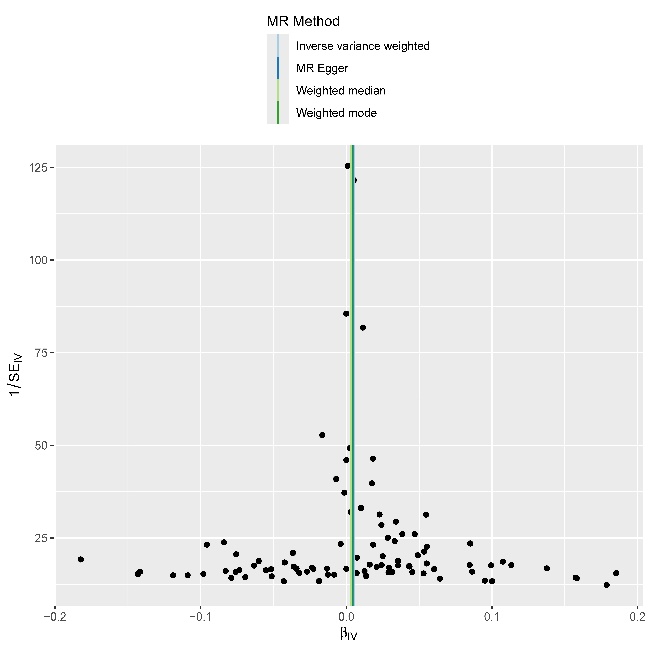


**Supplementary Figure 7**. Funnel plot of single-SNP effect estimates and corresponding inverse standard errors. **A)** coronary artery calcification and **B)** carotid intima-media thickness.

**
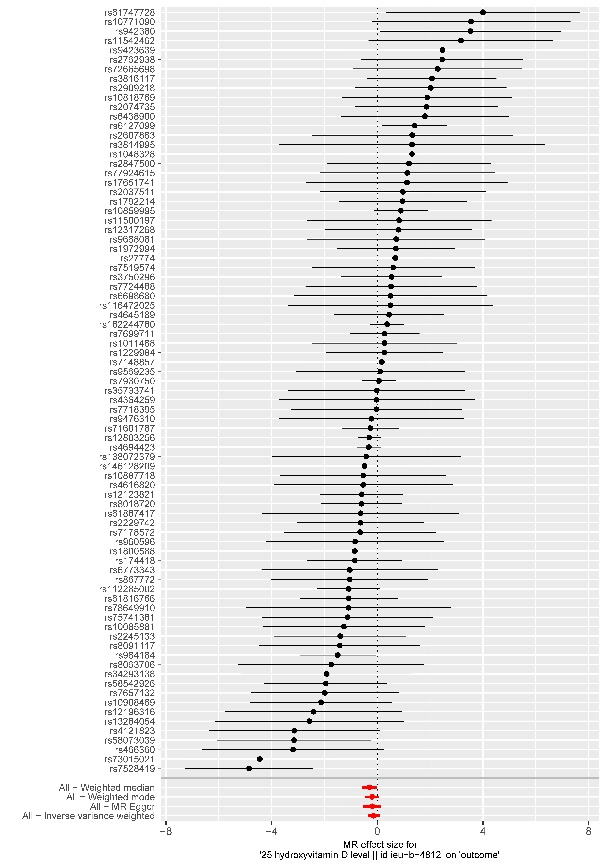

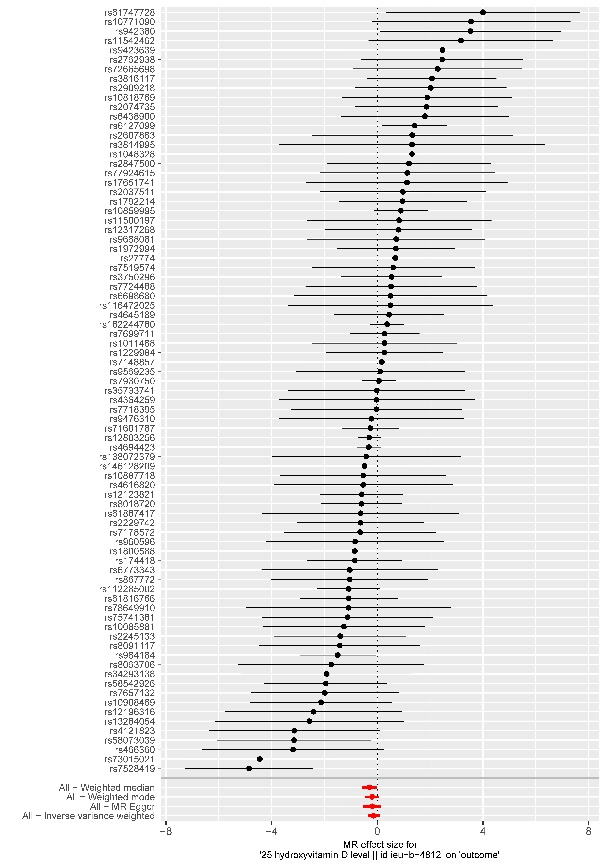
**

**B**

**A**

**Supplementary Figure 8**. Forest plots of the single and multi-SNP analyses for the SNP effect of vitamin D levels on **A)** coronary artery calcification and **B)** carotid intima-media thickness. The x-axis shows the MR effect size for the vitamin D levels on outcome of interest. The y-axis shows single-SNP (black lines) as well as multi-SNP ratio estimates (red line). Weighted median, weighted mode, and MR-Egger show sensitivity meta-analyses. Error bars indicate 95% confidence intervals.

**
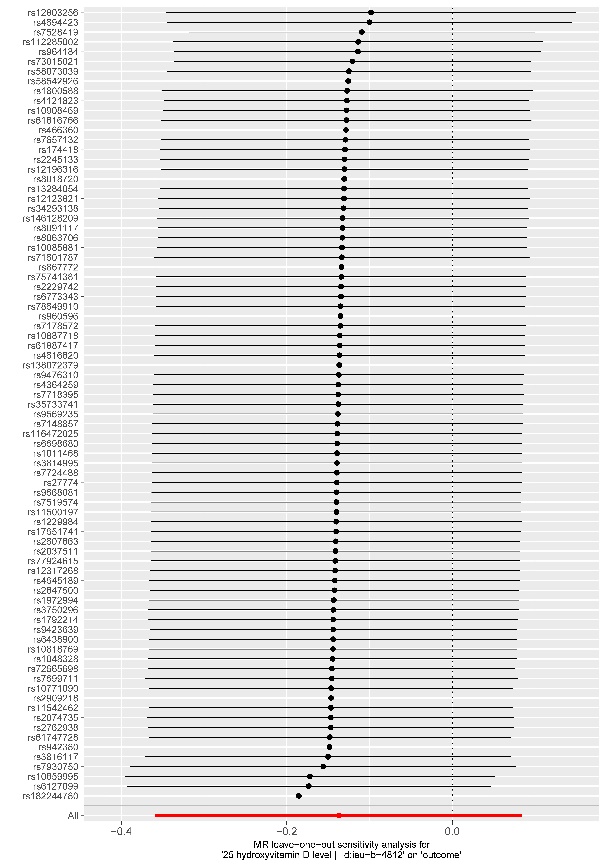

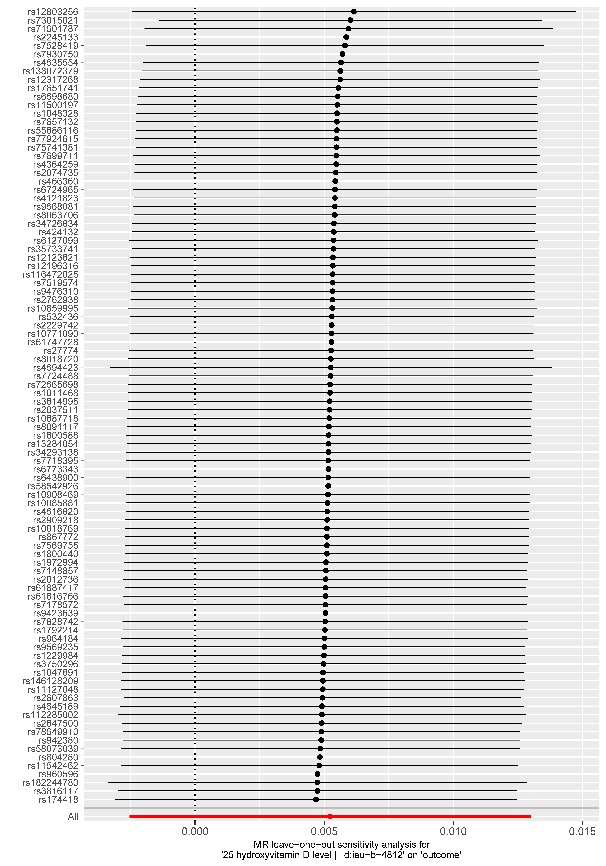
**

**B**

**A**

**Supplementary Figure 9**. Forest plot of "leave-one-out" sensitivity analysis method to show the influence of single-SNPs on the MR estimates. The black and red lines indicate single-SNP and multi-SNP analysis, respectively. Error bars indicate 95% confidence intervals. **A)** coronary artery calcification and **B)** carotid intima-media thickness.
